# Analysis of temporal transcription expression profiles reveal links between protein function and developmental stages of *Drosophila melanogaster*

**DOI:** 10.1371/journal.pcbi.1005791

**Published:** 2017-10-18

**Authors:** Cen Wan, Jonathan G. Lees, Federico Minneci, Christine A. Orengo, David T. Jones

**Affiliations:** 1 Department of Computer Science, University College London, London, United Kingdom; 2 Biomedical Data Science Laboratory, The Francis Crick Institute, London, United Kingdom; 3 Institute of Structural and Molecular Biology, University College London, London, United Kingdom; Bar Ilan University, ISRAEL

## Abstract

Accurate gene or protein function prediction is a key challenge in the post-genome era. Most current methods perform well on molecular function prediction, but struggle to provide useful annotations relating to biological process functions due to the limited power of sequence-based features in that functional domain. In this work, we systematically evaluate the predictive power of temporal transcription expression profiles for protein function prediction in *Drosophila melanogaster*. Our results show significantly better performance on predicting protein function when transcription expression profile-based features are integrated with sequence-derived features, compared with the sequence-derived features alone. We also observe that the combination of expression-based and sequence-based features leads to further improvement of accuracy on predicting all three domains of gene function. Based on the optimal feature combinations, we then propose a novel multi-classifier-based function prediction method for *Drosophila melanogaster* proteins, FFPred-fly+. Interpreting our machine learning models also allows us to identify some of the underlying links between biological processes and developmental stages of *Drosophila melanogaster*.

## Author summary

Despite painstaking experimental efforts and the extensive sequence similarity based annotation transfers, less than a half of the fruit fly protein sequences in UniProtKB have some functional annotation. To help fill in this gap, we test the usefulness of publicly available temporal gene expression profiles and their combination with many biophysical attributes that can be effectively derived from the corresponding protein sequence. We find that such an integrative function prediction method provides more accurate predictions than using sequence data alone and we expect these predictions to help narrow down the number of experimental assays required to characterise fly protein function. We demonstrate by highlighting correlations between predicted biological process functions and known facts about fly developmental stages.

## Introduction

Protein or gene function prediction is a difficult computational challenge which has received increasing attention in the previous decade, with one major goal being to assist experimental biologists in making testable hypotheses about the role of uncharacterised proteins in biological systems. *Ab initio* prediction of gene function using *in-silico* methods has made great strides in the recent years, with the best methods typically making use of various protein sequence-based features in a Machine Learning framework [1–4]. FFPred is one such method, as the main component method used by the Jones-UCL team, consistently ranked near the top in independent benchmark challenges [5, 6].

The most common method for predicting protein function is to rely on simple homology-based transfer, where function annotations are transferred from a well characterised protein to a target protein on the basis of clear common ancestry between the two. In contrast to methods exploiting direct homology-based information, FFPred predicts protein function using intrinsic features directly derived from protein sequence, such as amino acid composition, intrinsically disordered regions, signal peptides and so on. By using a wide variety of sequence features, FFPred shows better performance when making functional predictions for proteins where direct homology information provides little or no predictive power. However, while FFPred and other similar methods tend to perform well in molecular function prediction, the prediction accuracy for biological process function is frequently poorer. To assist in predicting biological process terms, it may be useful to integrate data that go beyond the features that can be derived solely from the protein sequence, such as RNA-seq data.

*Drosophila melanogaster* is a well-studied organism that is a common model used to investigate the complex biological mechanisms of development, such as cell migration, nervous system development and so forth. Therefore, there is value in developing a protein function prediction method, which aims to not only accurately predict protein function, but also be able to identify key biological processes associated with each developmental stage. To the best of our knowledge, there is no published work which systematically studies *Drosophila*-specific protein function prediction, except one relevant work done by Costello, et al. (2009) [7]. The authors proposed predicting *Drosophila* gene function by relying on gene networks that are constructed by integrating different data sources, such as microarray expression data, genetic interaction and protein-protein interactions. However, the authors did not study the additional predictive power of sequence information, which is the main data source in protein function prediction. They also only discuss the prediction of biological process terms, rather than terms from all three domains of function covered in this work. In addition, although some other existing protein function prediction methods, e.g. [8–10], show capacity to predict *Drosophila* protein function, all of them use models trained by integrating other species’ data, rather than specifically focusing on *Drosophila*, and none of them investigates the relationship between protein function and various developmental stages of the organism.

High coverage temporal transcription expression profile data already exists for *Drosophila* through modENCODE [11, 12]. The data include the time-course RNA expression information during the whole life-cycle of *Drosophila*. In contrast to the majority of tissue-specific [13, 14] or certain developmental stage-specific (such as embryo stage-specific [15, 16]) microarray gene expression data, this type of RNA-seq data provides the most complete gene expression information to help investigate the role of proteins played during the life-cycle of *Drosophila*. Using these datasets, we show we can improve the performance of protein function prediction in *Drosophila* and further discover informative links between protein function itself and *Drosophila* development.

In this work, we systematically evaluate the predictive power of temporal transcription expression profile data for protein function prediction. We firstly create FFPred-fly by re-training our standard FFPred model using *Drosophila*-specific sequence information, and then show how FFPred-fly can be combined with an RNA-seq dataset to significantly boost its performance in biological process function prediction. We choose *Drosophila* development as our exemplar RNA-seq dataset, so as to focus on a well-characterised developmental system, the results of which can be more readily interpreted. However, the framework we present is quite generic and could be easily extended to integrate FFPred with any organism specific RNA-seq dataset.

## Results

To carry out this study, we firstly generate new types of features according to the time-course transcription expression profiles obtained during the developmental stages of *Drosophila melanogaster*, i.e. the number (Num) of differently expressed transcripts on individual time-points, the average (Ave) expression profile on individual time-points over all transcripts of an individual gene, and the expression profile for the main-transcript (Main) of individual genes on individual time-points, and their different combinations, i.e. Num+Ave, Num+Main, Ave+Main and Num+Ave+Main. Note that, in this work, the main-transcript of one gene is defined as the protein isoform having the longest sequence among all isoforms, as suggested in [17]. In cases where more than two isoforms existing with the same length sequence, the transcript having the maximum expression value is selected as the main-transcript. We adopt the pre-processed source data that are identical to the ones used in [18].

**Fig 1:**
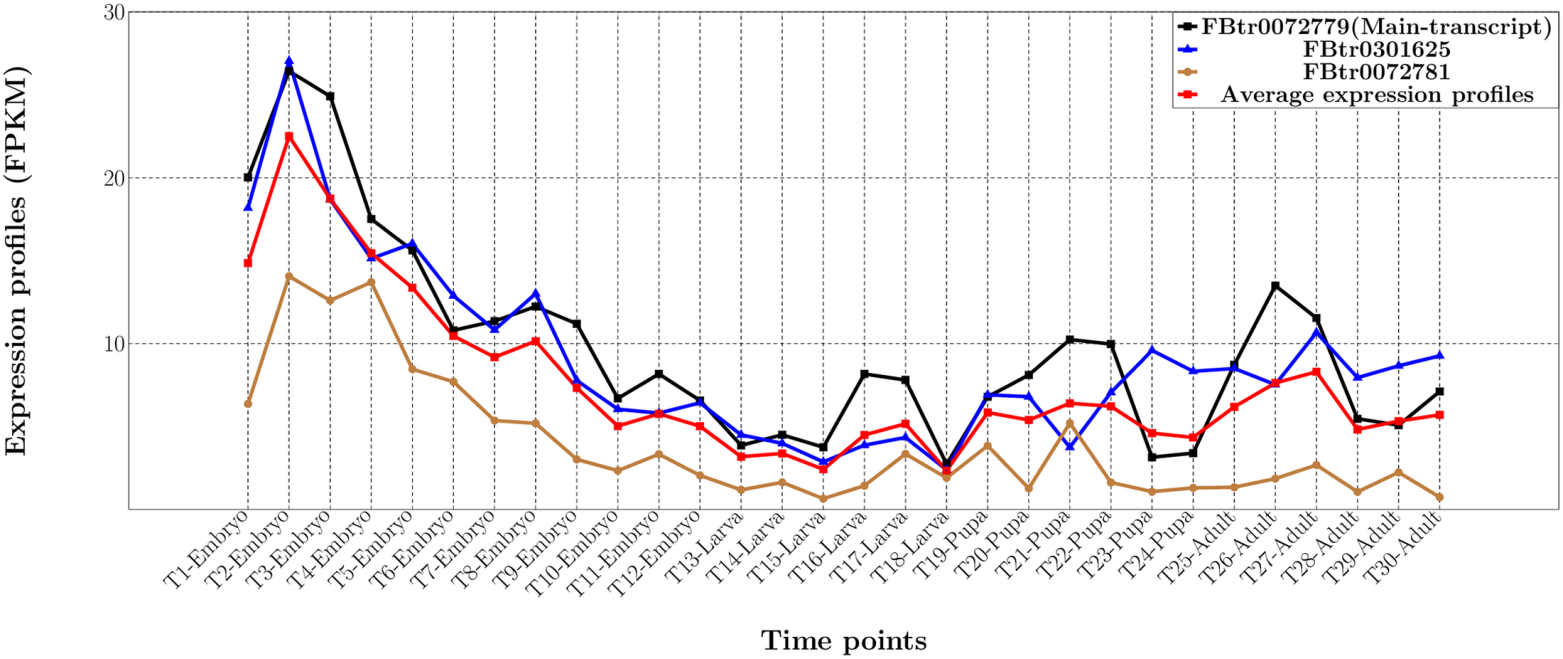
Expression profiles of three transcripts for gene FBgn0067864; the average expression profile over three transcripts (i.e. the red line); and the expression profile for the main-transcript (i.e. the black line).

Each type of feature consists of 30 individual variables denoting the transcription expression profile at 30 time-points, covering the four main developmental stages, i.e. embryo (T1—T12), larva (T13—T18), pupa (T19—T24) and adult (T25—T30). Fig 1 shows an example of all the transcripts’ expression profiles for gene FBgn0067864, i.e. the black line for FBtr0072779, the blue line for FBtr0301625 and the brown line for FBtr0072781. It is obvious that the expression profiles for all three transcripts are different at all individual time-points. Therefore, the values of Num type features are all equal to 3, since there exist three different transcripts for gene FBgn0067864 over all 30 time-points. The red line displays the values of the Ave type of features (i.e. average expression profile) for gene FBgn0067864 over three transcripts’ expression profiles; while the black line displays the values of the Main type of features, i.e. expression profile of the main-transcript FBtr0072779 for gene FBgn0067864.

Then we further combine these seven types of expression-based features with the conventional protein sequence-based (Seq) features, namely Seq+Num, Seq+Ave, Seq+Main, Seq+Num+Ave, Seq+Num+Main, Seq+Ave+Main and Seq+Num+Ave+Main. In order to evaluate the performance of these newly-generated features, we create protein sets for predicting 301 individual GO terms by adopting the consistent procedure described in [2]—see Materials and methods. Briefly, a set of proteins is created for each GO term, where a protein is labeled by a class denoting whether it is annotated with that GO term. We then conduct experiments on two types of protein sets: a) 7 protein sets exploiting only expression-based features to characterise the proteins; b) 7 protein sets exploiting the combination of expression-based and sequence-based features to characterise the proteins. The results of both types of protein sets are compared with the one obtained a protein set exploiting only the protein sequence-based features.

We firstly report the results for those datasets using four different classification algorithms, i.e. Random Forests (RF) [19]; Adaptive-Boosting (AdaBoost) [20]; k-Nearest Neighbours (KNN) [21]; and Linear Discriminant Analysis (LDA) [22] (see S1 Text). Note that, we also report the results obtained by the best-performing classification algorithm (Opt-Classifier) for each GO term, in order to alleviate the bias on choosing different classification algorithms. Then we report the results for evaluating our newly-proposed *Drosophila*-specific protein function prediction method, FFPred-fly+, exploits the best-performing types of features for individual GO term domains.

### Transcription expression profile-based features show improved accuracy for predicting biological process function

We firstly evaluate the predictive power of individual types of expression-based features by comparing with the sequence-based features. Tables 1 and 2 report the mean MCC and AUROC values obtained by predicting different domains of GO terms using four different classification algorithms and additionally the Opt-Classifier. The bold-type figures denote the highest mean MCC or AUROC value for each column.

**Table 1:** Mean MCC values obtained by different expression-based feature groups and the sequence-based feature group over cross validation.

Feature Group	RF			AdaBoost			KNN			LDA			Opt–Classifier		
	BP	MF	CC	BP	MF	CC	BP	MF	CC	BP	MF	CC	BP	MF	CC
**Num**	0.065	0.119	0.081	0.044	0.082	0.049	0.060	0.085	0.070	0.029	0.019	0.029	0.092	0.129	0.095
**Ave**	**0.254**	0.301	0.354	0.186	0.240	0.250	0.229	0.267	0.327	0.048	0.062	0.114	**0.268**	0.309	0.364
**Main**	0.236	0.270	0.340	0.163	0.214	0.231	0.224	0.244	0.319	0.045	0.059	0.115	0.254	0.281	0.350
**Num+Ave**	0.249	0.295	0.349	0.190	0.249	0.249	0.231	0.266	0.321	0.069	0.072	0.130	0.267	0.310	0.359
**Num+Main**	0.228	0.278	0.333	0.169	0.223	0.241	0.220	0.252	0.308	0.067	0.067	0.134	0.248	0.293	0.340
**Ave+Main**	0.247	0.285	0.347	0.190	0.245	0.252	**0.232**	0.261	0.325	0.055	0.068	0.128	0.264	0.297	0.360
**Num+Ave+Main**	0.247	0.292	0.345	**0.192**	0.245	0.250	0.229	0.264	0.314	0.076	0.077	0.139	0.264	0.308	0.355
**Seq**	0.196	**0.485**	**0.366**	0.173	**0.466**	**0.367**	0.203	**0.449**	**0.365**	**0.177**	**0.454**	**0.381**	0.239	**0.519**	**0.411**

**Table 2:** Mean AUROC values obtained by different expression-based feature groups and the sequence-based feature group over cross validation.

Feature Group	RF			AdaBoost			KNN			LDA			Opt–Classifier		
	BP	MF	CC	BP	MF	CC	BP	MF	CC	BP	MF	CC	BP	MF	CC
**Num**	0.583	0.598	0.596	0.580	0.589	0.592	0.555	0.571	0.568	0.578	0.593	0.589	0.597	0.612	0.608
**Ave**	0.698	0.743	0.785	0.683	0.713	0.763	0.692	0.731	0.771	0.632	0.678	0.723	0.709	0.750	0.795
**Main**	0.678	0.720	0.777	0.654	0.692	0.751	0.671	0.711	0.761	0.619	0.668	0.715	0.688	0.728	0.786
**Num+Ave**	**0.700**	0.741	0.787	**0.684**	0.717	0.766	**0.695**	0.730	0.774	**0.636**	0.675	0.708	**0.712**	0.749	0.794
**Num+Main**	0.681	0.723	0.774	0.663	0.700	0.753	0.681	0.719	0.763	0.630	0.670	0.704	0.695	0.734	0.782
**Ave+Main**	0.698	0.736	0.788	0.680	0.710	0.763	0.689	0.721	0.771	0.629	0.677	0.724	0.707	0.742	0.796
**Num+Ave+Main**	0.699	0.738	0.784	0.682	0.712	0.764	0.693	0.725	0.772	0.635	0.677	0.709	0.710	0.745	0.793
**Seq**	0.666	**0.849**	**0.801**	0.649	**0.821**	**0.792**	0.665	**0.819**	**0.803**	0.632	**0.813**	**0.784**	0.653	**0.855**	**0.817**

In general, for predicting biological process function, expression-based features give the higher accuracy compared with the sequence-based features. Three of the four classification algorithms obtain the higher mean MCC values by adopting expression-based features (i.e. RF with Ave, AdaBoost with Num+Ave+Main and KNN with Ave+Main), while all four types of classification algorithms obtain the higher mean AUROC values by using Num+Ave features. In addition, the highest result obtained by the Opt-Classifier (i.e. 0.268 of the mean MCC value obtained by Ave and 0.712 of the mean AUROC value obtained by Num+Ave) also suggests the better predictive performance of expression-based features.

In terms of predicting molecular function and cellular component terms, sequence-based features give higher mean MCC values and AUROC values, when using all four types of classification algorithms. This fact is further confirmed with the results obtained by the Opt-Classifier, i.e. 0.519 and 0.411 of the mean MCC value, 0.855 and 0.817 of the mean AUROC value, respectively for MF and CC terms.

**Fig 2:**
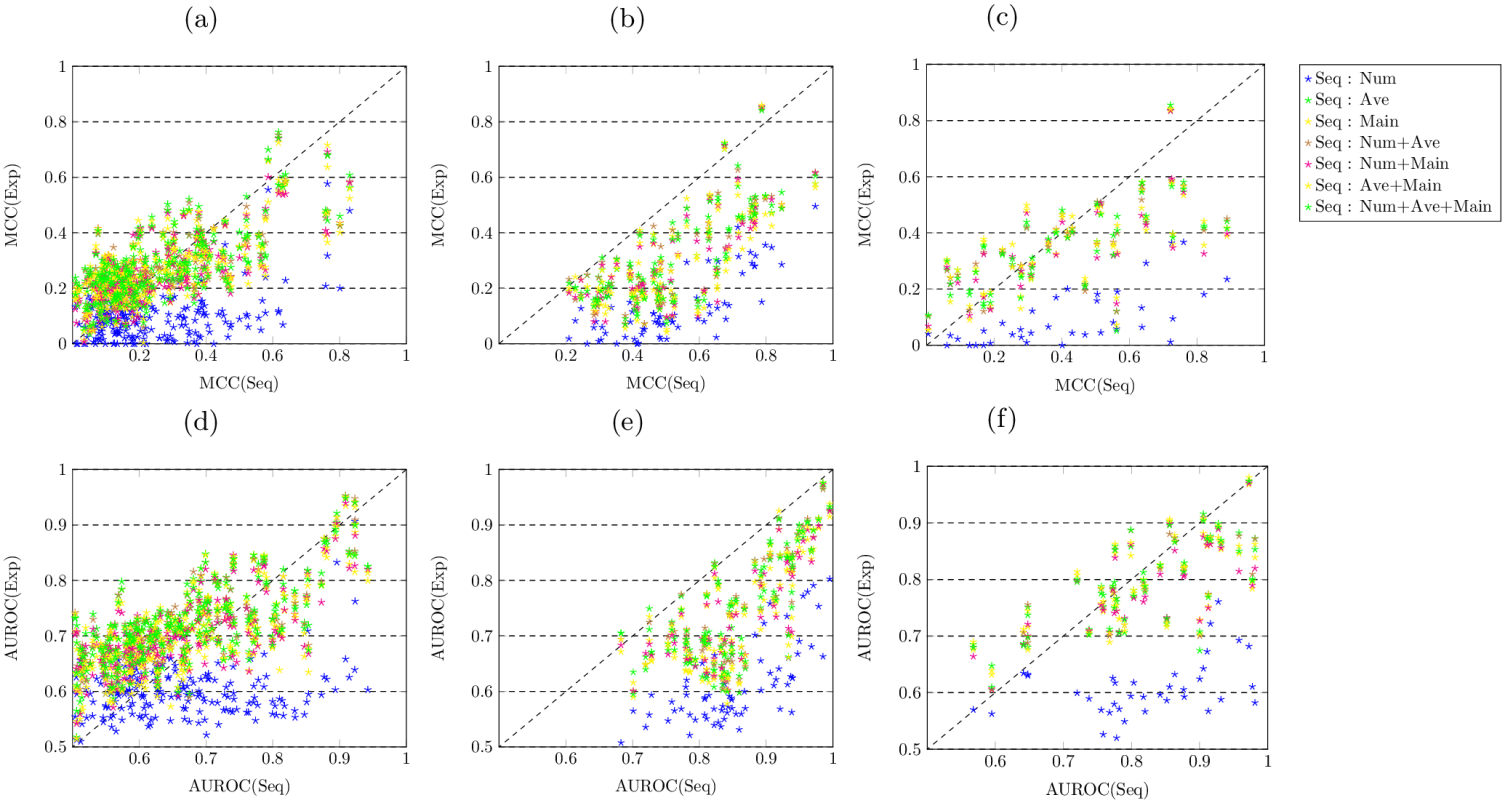
Expression-based features show competitive performance against the sequence-based features for predicting the biological process domain of protein function. (a,d) the MCC and AUROC values obtained by different features for predicting the biological process domain of GO terms over cross validation. (b,e) the MCC and AUROC values obtained by different features for predicting the molecular function domain of GO terms over cross validation. (c,f) the MCC and AUROC values obtained by different features for predicting the cellular component domain of GO terms over cross validation.

We then report the MCC and AUROC values obtained by all different types of features when predicting all 301 GO terms. The scatter plots in Fig 2.a, 2.b, 2.c, 2.d, 2.e and 2.f respectively display the MCC and AUROC values obtained by the Opt-Classifier for predicting three individual domains of protein function. In each scatter plot, the x axis represents the MCC or AUROC values obtained by the sequence-based features, while the y axis represents the MCC or AUROC values obtained by different types of expression-based features. The red diagonal indicates the case when the MCC or AUROC values obtained by the sequence-based features and individual type of expression-based features are equal. The different colours of dots denote the pairs of MCC or AUROC values obtained by different types of expression-based features and the sequence-based features.

In terms of predicting biological process function, as shown in Fig 2.a, the dots in different colours (except blue) drop on both sides of the diagonal, while the blue dots almost all fall into the area below the diagonal. In Fig 2.d, the majority of dots in different colors (except blue) fall on the area above the diagonal. This fact indicates that all types of expression-based features (except Num) obtain better performance than the sequence-based features. For predicting molecular function, as shown in Fig 2.b and 2.e, almost all dots drop in the area below the diagonal, indicating the consistent fact that all types of expression-based features perform worse than the sequence-based features. For predicting cellular component function, as shown in Fig 2.c and 2.f, all dots in different colours (except blue) drop on the both sides of the diagonal. It suggests that expression-based features (except the Num) show similar predictive performance against the sequence-based features.

We conduct the Wilcoxon signed-rank test (two-tailed at 5% of significance level) on each pair of the MCC or AUROC values obtained by the individual types of expression-based features and sequence-based features. The results are included in Tables A and B in S1 Text. Overall, the significance test results confirm the findings. To begin with, one type of expression-based features—Num, performs significantly worse than the sequence-based features when predicting all three domains of protein function. Moreover, for predicting biological process function, all types of expression-based features (except Num) significantly outperform the sequence-based features, with one exception of the Num+Main features obtaining non-significantly better MCC values than the sequence-based features. Furthermore, for predicting molecular function, all types of expression-based features perform worse than sequence-based features. For predicting cellular component function, almost all types of expression-based features (except Num) perform non-significantly differently to sequence-based features, with exceptions of the Main and Num+Main features. The former obtains a significantly lower MCC value, while the latter obtains significantly lower MCC and AUROC values, compared with the Seq type of features.

### Combining expression-based and sequence-based features further improves the accuracy for predicting all three domains of protein function

We further evaluate the predictive power of the combination of expression-based and sequence-based features. We report the mean MCC and AUROC values obtained by different types of feature combinations in Tables 3 and 4. Overall, the combinations of expression-based and sequence-based features obtain higher mean MCC and AUROC values when predicting all three domains of protein function. Almost all of the four classification algorithms obtain higher mean MCC and AUROC values by exploiting the feature combinations for predicting the three domains of protein function, except the KNN classification algorithm, which obtains better results by only adopting sequence-based features for molecular function and cellular component function prediction.

**Table 3:** Mean MCC values obtained by different combinations of expression-based and the sequence-based feature groups over cross validation.

Feature Group	RF			AdaBoost			KNN			LDA			Opt–Classifier		
	BP	MF	CC	BP	MF	CC	BP	MF	CC	BP	MF	CC	BP	MF	CC
**Seq**	0.196	0.485	0.366	0.173	0.466	0.367	0.203	**0.449**	**0.365**	0.177	0.454	0.381	0.239	0.519	0.411
**Seq+Num**	0.202	0.497	0.369	0.180	0.470	0.369	0.173	0.369	0.313	0.181	0.457	0.384	0.235	0.516	0.408
**Seq+Ave**	0.230	0.498	0.400	0.214	0.486	0.410	0.226	0.305	0.336	0.189	0.466	0.394	0.279	0.527	0.452
**Seq+Main**	0.217	0.499	0.407	0.201	0.484	0.411	0.218	0.287	0.339	0.189	0.466	0.394	0.274	0.522	0.449
**Seq+Num+Ave**	0.234	**0.507**	0.400	0.216	0.488	0.408	0.228	0.288	0.331	0.194	0.467	0.395	0.285	0.528	0.452
**Seq+Num+Main**	0.224	0.497	0.402	0.206	0.487	0.405	0.220	0.278	0.324	0.192	0.466	0.397	0.279	0.526	0.447
**Seq+Ave+Main**	0.239	0.502	**0.410**	0.217	**0.490**	**0.418**	**0.230**	0.290	0.336	0.193	**0.472**	0.397	0.286	**0.530**	**0.463**
**Seq+Num+Ave+Main**	**0.244**	0.493	0.409	**0.218**	**0.490**	0.417	0.227	0.282	0.322	**0.197**	0.471	**0.399**	**0.287**	0.526	0.461

**Table 4:** Mean AUROC values obtained by different expression-based feature groups and the sequence-based feature group over cross validation.

Feature Group	RF			AdaBoost			KNN			LDA			Opt–Classifier		
	BP	MF	CC	BP	MF	CC	BP	MF	CC	BP	MF	CC	BP	MF	CC
**Seq**	0.666	0.849	0.801	0.649	0.821	0.792	0.665	**0.819**	**0.803**	0.632	0.813	0.784	0.653	0.855	0.817
**Seq+Num**	0.671	0.848	0.804	0.654	0.823	0.796	0.648	0.769	0.776	0.636	0.814	0.786	0.685	0.852	0.814
**Seq+Ave**	0.696	0.856	0.835	0.679	0.831	0.819	**0.693**	0.743	0.778	0.642	0.821	0.791	0.721	0.860	0.845
**Seq+Main**	0.687	0.856	0.828	0.665	0.830	0.815	0.678	0.727	0.771	0.641	0.820	0.791	0.712	0.860	0.840
**Seq+Num+Ave**	0.701	**0.858**	0.835	0.679	**0.832**	0.819	0.692	0.734	0.777	0.645	0.821	0.793	0.679	**0.862**	0.845
**Seq+Num+Main**	0.692	0.855	0.830	0.668	0.831	0.816	0.682	0.724	0.770	0.644	0.820	0.793	0.716	0.858	0.841
**Seq+Ave+Main**	0.703	0.857	**0.842**	0.680	0.831	0.819	0.690	0.731	0.776	0.646	**0.824**	0.792	0.722	0.861	**0.848**
**Seq+Num+Ave+Main**	**0.709**	0.855	**0.842**	**0.682**	0.831	**0.820**	0.692	0.730	0.775	**0.649**	**0.824**	**0.794**	**0.726**	0.859	**0.848**

The Opt-Classifier also obtains the highest mean MCC and AUROC values for predicting the three domains of function by exploiting the combinations of feature types (i.e. MCC of 0.287 and AUROC of 0.726 for predicting BP terms with Seq+Num+Ave+Main features; MCC of 0.530 with Seq+Ave+Main features and AUROC of 0.862 with Seq+Num+Ave features for predicting MF terms; MCC of 0.463 with Seq+Ave+Main features and AUROC of 0.848 with either Seq+Ave+Main or Seq+Num+Ave+Main features for predicting CC terms).

**Fig 3:**
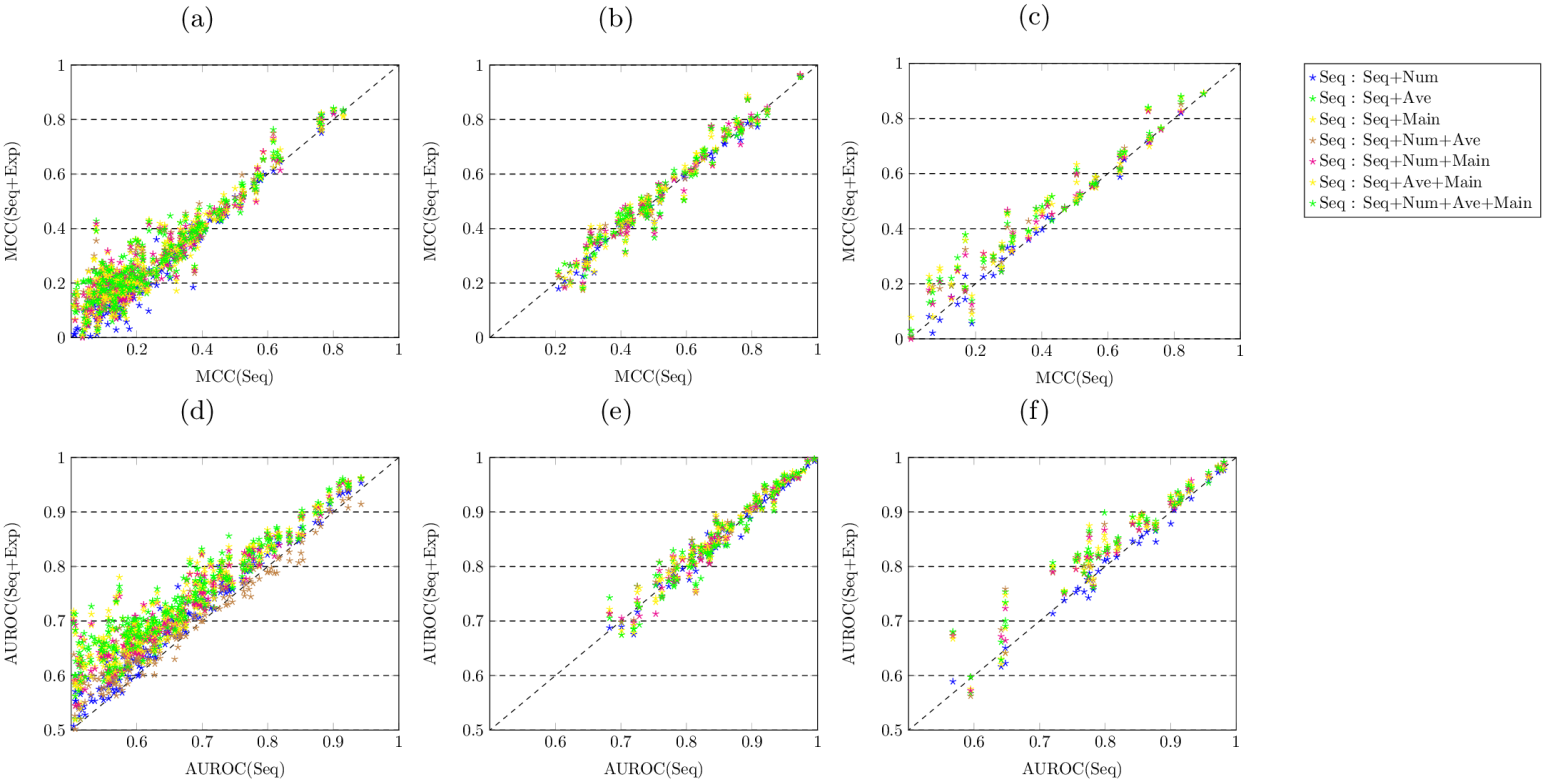
Combining expression-based features with sequence-based features boosts the predictive accuracy when only adopting sequence-based features for predicting all three domains of protein function. (a,d) the MCC and AUROC values obtained by different features for predicting biological process domain of GO terms over cross validation. (b,e) the MCC and AUROC values obtained by different features for predicting molecular function domain of GO terms over cross validation. (c,f) the MCC and AUROC values obtained by different features for predicting cellular component domain of GO terms over cross validation.

We also report the MCC and AUROC values obtained by predicting all GO terms with the Opt-Classifier. Analogously to Fig 2, the scatter plots in Fig 3 display the comparison of MCC and AUROC values obtained by the sequence-based features and its combination with different types of expression-based features. For predicting biological process function, as shown in Fig 3.a and 3.d, the majority of dots drop in the area above the diagonal. This suggests the fact that merging expression-based features with sequence-based features improves the predictive performance, compared with only adopting the sequence-based features. For predicting molecular function, as shown in Fig 3.b and 3.e, almost all dots drop on both sides of the diagonal, indicating similar predictive power of sequence-based features and its combinations with expression-based features. For predicting cellular component function, the combinations of expression-based and sequence-based features outperform sequence-based features, since the majority of plots drop in the area above the diagonal.

The Wilcoxon signed-rank test results also confirm that all combinations of expression-based and sequence-based features (except Seq+Num) obtain significantly higher accuracy than only sequence-based features for predicting both BP and CC domains of protein function. In the case of predicting molecular function, almost all combinations of expression-based and sequence-based features show non-significant differences except feature combinations Seq+Num+Ave and Seq+Ave+Main which both show significantly higher MCC values, while all feature combinations obtain significantly higher AUROC values except Seq+Num and Seq+Num+Ave+Main.

### The optimal features and best performing classification algorithm for predicting three domains of protein function

We further compare the predictive accuracy obtained by all 15 different types of features over the cross validation procedure. The results are shown in the boxplots in Figures A and B in S1 Text. Overall, the Seq+Num+Ave+Main type of features obtains the best accuracy (also obtains the best ranking by considering both MCC and AUROC values, as shown in Table C in S1 Text) for predicting biological process function, whereas both the Seq+Num+Ave and Seq+Ave+Main features performs best for predicting molecular function terms. Seq+Ave+Main features also performs the best for predicting cellular component function. We then further compare the predictive performance of different types of features using a larger training protein set, i.e. adopting the whole 70% of the protein set for training, then testing on the remaining 30% of protein set. The results show that Seq+Num+Ave+Main features perform best for predicting BP and MF domains of protein function, while the Seq+Ave+Main features obtain the highest accuracy on predicting cellular component function, since Seq+Num+Ave+Main and Seq+Ave+Main features respectively obtain the best ranking for predicting corresponding domains of protein function, by considering both MCC and AUROC values, as shown in Table D in S1 Text.

We also compare the predictive performance of different classification algorithms. The pie-charts in Figures C and D in S1 Text display the proportion of GO terms for which individual classification algorithm obtains the best performance. In general, the RF is the best performing classification algorithm. For predicting biological process function, KNN and RF are the best performing classification algorithms, but RF outperforms other classification algorithms on predicting other two domains of function.

### FFPred-fly+, a novel protein function prediction method for *Drosophila melanogaster*

We further propose a new *Drosophila melanogaster*-specific protein function prediction method, namely FFPred-fly+, by exploiting the optimal combination of expression-based and sequence-based features w.r.t. corresponding domain of protein function. According to the results discussed in the previous section, we use Seq+Num+Ave+Main features for predicting biological process function and molecular function, and Seq+Ave+Main features for predicting cellular component function. FFPred-fly+ considers 4 different candidate classification algorithms (i.e. RF, AdaBoost, KNN and LDA). It firstly selects the single best classification algorithm for each GO term according to the predictive performance on cross validation with varying numbers of splits of the training set, depending on the number of proteins with that GO term (see [2] for details). Then the selected algorithm is trained on the whole protein training set. The performance of trained classifier is evaluated by conducting the prediction on the independent protein test set. Note that, in [2], the performance of FFPred-fly was evaluated by testing on a 30% split test set. Hence, in this work, we evaluate the relative performance of FFPred-fly+ by testing on the same 30% test set, while conducting the classification algorithm selection and training process on a 70% split as the training set. In other words, the training data is used for both algorithm selection and training, but not for final testing.

**Fig 4:**
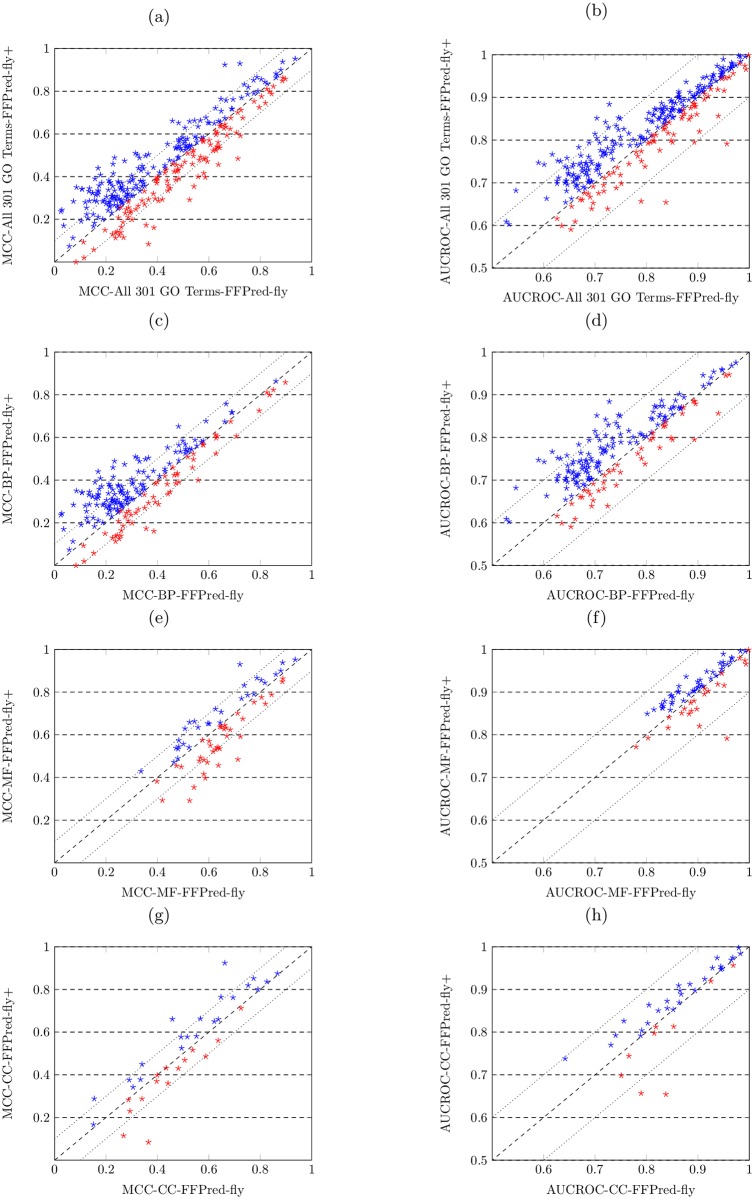
FFPred-fly+ shows better performance than FFPred-fly when predicting all three domains of protein function. (a,b) FFPred-fly+ obtains higher MCC and AUROC values on predicting all 301 GO terms; (c,d) FFPred-fly+ obtains higher MCC and AUROC values on predicting 196 biological process GO terms; (e,f) FFPred-fly+ obtains similar MCC values but higher AUROC values on predicting 68 molecular function GO terms; (g,h) FFPred-fly+ obtains similar MCC values but higher AUROC values on predicting 37 cellular component GO terms.

The results are shown in the scatter plots in Fig 4, where the MCC and AUROC values obtained by both methods are displayed. In each figure, the values on x-axis denotes the MCC or AUROC values obtained by the FFPred-fly approach, while the values on y-axis denote the MCC or AUROC values obtained by the FFPred-fly+ approach; the diagonal indicates the case when the MCC or AUROC values for the prediction on same function obtained by two approaches are equal; the plots in blue indicate the MCC or AUROC values obtained by the FFPred-fly+ are greater than the ones obtained by the FFPred-fly. Two dashed lines on both of sides of diagonal indicate the value of difference on MCC or AUROC values obtained by two approaches is 0.1.

Overall, for predicting all 301 GO terms on the three different domains of protein function, as shown in Fig 4.a and 4.b, FFPred-fly+ outperforms FFPred-fly, since more dots drop in the area above the diagonal. In detail, 187 out of 301 GO terms obtain higher MCC values by using FFPred-fly+. Among those 187 function, the difference in MCC values obtained by two approaches is greater than 0.1 for 56 GO terms. Among those 114 of functions obtained higher MCC values by FFPred-fly, the difference in MCC values obtained by two approaches is greater than 0.1 for merely 27 GO terms. Analogously, 230 out of 301 GO terms obtain higher AUROC values by using FFPred-fly+. 40 GO terms obtain 0.1 higher AUROC values with FFPred-fly+, whereas only 3 GO terms obtain 0.1 higher AUROC values with FFPred-fly.

FFPred-fly+ performs better on predicting biological process function, as shown in Fig 4.c and 4.d, most of the dots in blue drop on the area above the diagonal. In detail, FFPred-fly+ obtains higher MCC values for 135 out of 196 BP terms, while 47 of them have 0.1 higher MCC values than the ones obtained by FFPred-fly. 157 out of 196 BP terms obtained higher AUROC values by FFPred-fly+, while 40 of them have 0.1 higher than the ones obtained by FFPred-fly.

For predicting molecular function, as shown in Fig 4.e and 4.f, FFPred-fly+ and FFPred-fly show comparable predictive performance, since the numbers of blue and red dots are similar. The latter obtains higher MCC values on slightly more terms (i.e. 37 out of 68), whereas the former obtains higher AUROC values on more terms (i.e. 45 out of 68). Among those 37 MF terms with higher MCC values, 10 terms’ MCC values are 0.1 higher than the ones obtained by FFPred-fly+. Among those 31 of GO terms with higher MCC values obtained by FFPred-fly+, 4 terms’ MCC values are 0.1 higher than the ones obtained by FFPred-fly.

For predicting cellular component function, as shown in Fig 4.g and 4.h, FFPred-fly+ shows better predictive performance. FFPred-fly+ obtains higher MCC values for 22 out of 37 GO terms and higher AUROC values for 28 out of 37 terms. 5 of 22 terms are 0.1 higher MCC values to the ones obtained by FFPred-fly. 3 of 15 terms obtain 0.1 higher MCC values by FFPred-fly, and 2 out of 9 terms higher AUROC values, compared with the ones obtained by FFPred-fly+.

We also conduct the Wilcoxon signed-ranked test (two-tailed at 5% significance level) on MCC and AUROC values obtained by the two approaches. The results of significance test on MCC values suggest that FFPred-fly+ significantly outperforms FFPred-fly when predicting 196 BP function (p-value(BP) = 9.6e-08), whereas no significant difference between the performance of two approaches when predicting MF and CC function. Conversely, FFPred-fly+ obtains significantly better AUROC values when predicting all GO terms (p-value(All)<2.2e-16), but also for predicting the individual domains of GO, i.e. p-value(BP)<2.2e-16, p-value(MF) = 9.0e-03 and p-value(CC) = 2.8e-02.

Table 5 displays 15 GO terms (5 for each domain of protein function) that obtain the biggest improvement on predictive performance by FFPred-fly+, compared with FFPred-fly. The meanings of the first 7 columns are self-explanatory, while the rightmost column of $\sqrt{\left(MCC\times Inc\right)}$ is a normalised value by simultaneously considering the actual MCC value and the increase on MCC obtained by FFPred-fly+. All GO terms in Table 5 in each domain are ranked in descending order according to the values of $\sqrt{(MCC\times Inc}$. Generally, for predicting BP terms, GO:0007051 obtains the highest $\sqrt{\left(MCC\times Inc\right)}$ value. The MCC value obtained for GO:0007051 reaches 0.489 (with Random Forests) with an increase of 0.256, while the MCC value obtained by FFPred-fly is 0.233. For predicting MF terms, the MCC value for the top-ranked term, i.e. GO:0003735, reaches 0.930 (which is also the highest among all MCC values obtained by predicting MF terms shown in this table), with an increase of 0.209. For predicting CC terms, the MCC value for the top-ranked term GO:0005840 reaches 0.924 (which is also the highest among all MCC values obtained by predicting CC domain of terms shown in this table), with an increase of 0.262.

**Table 5:** GO terms obtained most improvement on predictive performance by FFPred-fly+.

GO_ID	Name	Category	Opt–Classifier	MCC–FFPred-fly+	MCC–FFPred-fly	Increase	$\sqrt{\left(MCC\times Inc\right)}$
GO:0007051	spindle organization	BP	RF	0.489	0.233	0.256	0.354
GO:0022604	regulation of cell morphogenesis	BP	KNN	0.440	0.169	0.271	0.345
GO:0008380	RNA splicing	BP	LDA	0.652	0.482	0.170	0.333
GO:0007268	chemical synaptic transmission	BP	KNN	0.459	0.227	0.232	0.326
GO:0000278	mitotic cell cycle	BP	ADB	0.510	0.314	0.196	0.316
GO:0003735	structural constituent of ribosome	MF	ADB	0.930	0.721	0.209	0.441
GO:0003676	nucleic acid binding	MF	RF	0.659	0.525	0.134	0.297
GO:0008270	zinc ion binding	MF	RF	0.663	0.544	0.119	0.281
GO:0015267	channel activity	MF	RF	0.832	0.738	0.094	0.280
GO:0003677	DNA binding	MF	RF	0.629	0.507	0.122	0.277
GO:0005840	ribosome	CC	RF	0.924	0.662	0.262	0.492
GO:0030529	intracellular ribonucleoprotein complex	CC	LDA	0.661	0.458	0.203	0.366
GO:0005789	endoplasmic reticulum membrane	CC	RF	0.764	0.647	0.117	0.299
GO:0005615	extracellular space	CC	RF	0.852	0.774	0.078	0.258
GO:0005634	nucleus	CC	RF	0.663	0.567	0.096	0.252

**Fig 5:**
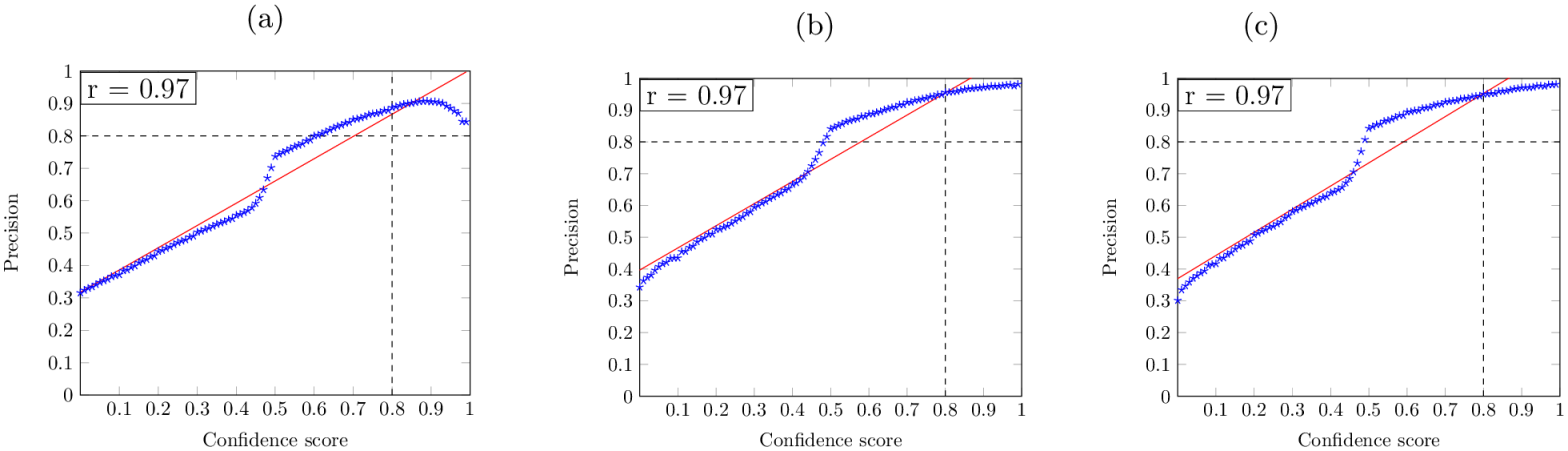
Correlation coefficient between the predictive confidence score and precision value for predicting BP (a), MF (b) and CC (c) terms.

We then further evaluate the reliability of prediction confidence score estimation by FFPred-fly+. Here we define the prediction confidence score as the posterior probability of the predicted GO term annotation for each protein. The higher the confidence score, the higher the likelihood that the annotation is correct. We calculate the correlation coefficient between the varying confidence score thresholds and the corresponding precision values. In more detail, we define a positive prediction if the prediction’s confidence score is greater than the given confidence score threshold. Then the precision value is calculated by $\frac{TP}{TP+FP}$, where *TP* denotes the numbers of corrected predicted annotations and *TP* + *FP* denotes the numbers of all predictions, where the confidence score is greater than the threshold. As shown in Fig 5, the confidence score for predicting all three domains of GO terms shows a positive correlation with the precision value (the r values are nearly equal to 1.00). For example, the precision values are all greater than 0.8 for predicting BP terms when the threshold is greater than 0.8. This further confirms that FFPred-fly+ is able to make good confidence score estimates for the predicted GO term annotations.

We then further verify the predicted protein-GO term annotations by screening the false positive predicted proteins by FFPred-fly+. The new protein-GO term annotations are generated by adopting new versions of the data (GOA.gaf 03-10-2016 & go.obo 08-10-2016). Table 6 displays 5 examples of predicted proteins included in Swiss-Prot and annotated with the GO terms by merely experimental evidence codes (e.g. IMP). The rightmost column denotes the number of ancestor layers in the GO hierarchy for individual GO terms. Note that, according to the GO hierarchy, the more ancestor layers the GO term has, the more specific the protein function that GO term defines. Our system successfully detects proteins’ annotations of GO terms with 6 or 7 ancestor layers. For example, protein RE32936p is successfully predicted as relating to “generation of neurons” (GO:0048699); “protein Box A-binding factor” is successfully predicted as involved with the process of animal organ morphogenesis (GO:0009887).

**Table 6:** Validated proteins predicted by FFPred-fly+.

Protein Description	Gene	GO_ID	GO Description	No. *Π*
RE32936p	Smyd4-2	GO:0048699	generation of neurons	7
Box A-binding factor	srp	GO:0009887	animal organ morphogenesis	6
RING finger protein unkempt	unk	GO:0022008	neurogenesis	6
Chromatin-remodeling complex ATPase chain Iswi	Iswi	GO:0007281	germ cell development	6
Eukaryotic translation initiation factor 3 subunit B	eIF3-S9	GO:0007292	female gamete generation	6

### FFPred-fly+ performs better than using InterPro for protein function prediction

The InterPro database [23] includes functional information on families, domains and other descriptive information about each protein. In this work, we compare our FFPred-fly+ method with an InterPro-based method for predicting those same 301 GO terms. We firstly obtain the domain, families and other protein descriptive information for 39855 *Drosophila* proteins by accessing the *match_complete.xml* file (release date 09/07/2014). Then we assign the GO term annotations to each protein through the *interpro2go* file (release date 04/07/2014).

**Fig 6:**
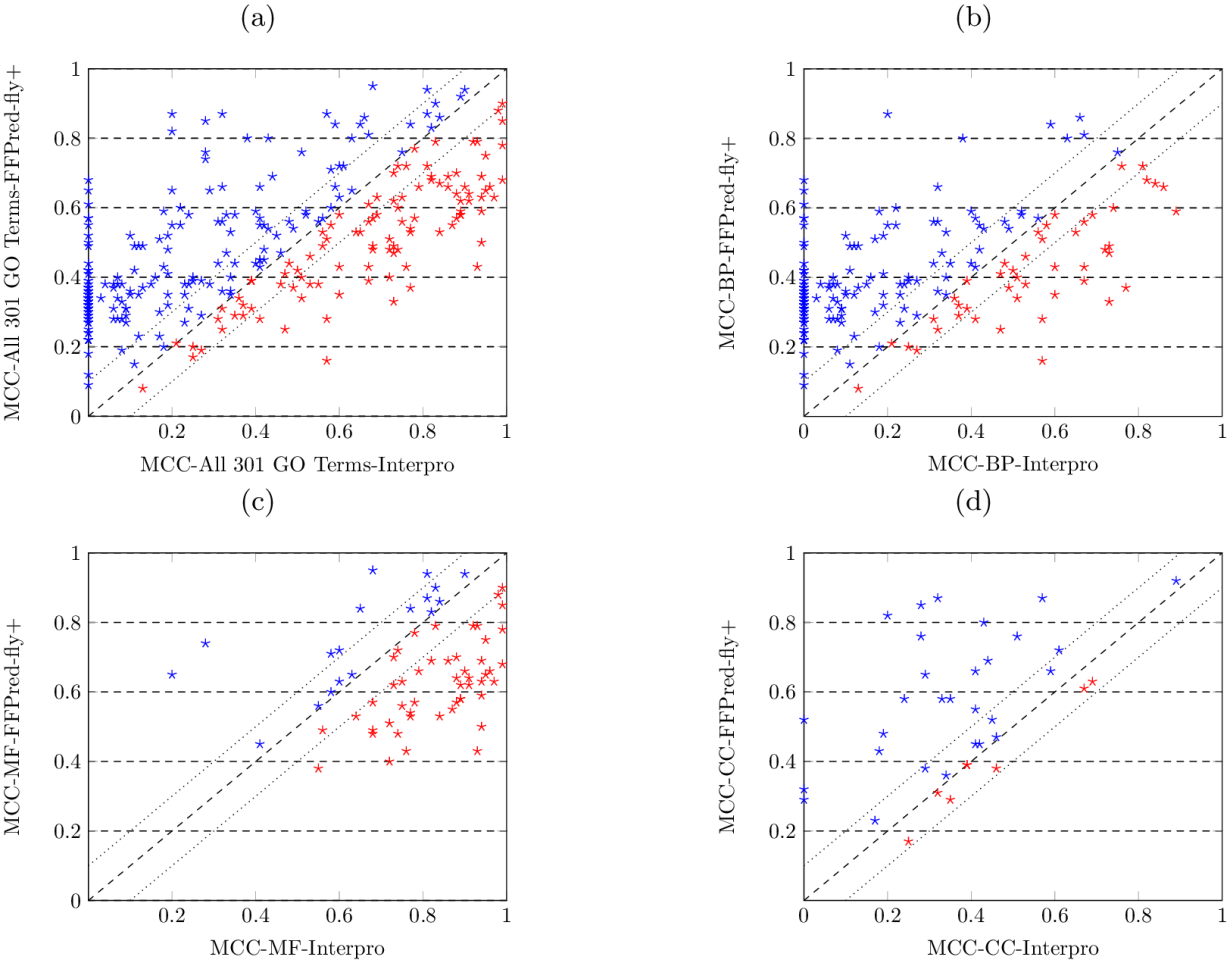
FFPred-fly+ shows higher predictive accuracy comparing with using InterPro database for predicting biological process and cellular component domains of protein function. (a) FFPred-fly+ obtains higher MCC values on predicting all 301 GO terms; (b) FFPred-fly+ obtains higher MCC values on predicting 196 biological process domain of GO terms; (c) Using InterPro database obtains better MCC values on predicting 68 molecular function domain of GO terms; (d) FFPred-fly+ obtains higher MCC values on predicting 37 cellular component domain of GO terms.

Overall, FFPred-fly+ performs better, especially when predicting biological process and cellular component function terms, whereas InterPro performs better for predicting molecular function terms. The results are shown in Fig 6, where the x-axis denotes the MCC values obtained by using InterPro, and the y-axis denotes the MCC values obtained by FFPred-fly+. In detail, when predicting all 301 GO terms, FFPred-fly+ obtains higher MCC values for 197 terms, while 158 of them exceed the MCC score obtained using InterPro by more than 0.1. Note that, 72 terms obtain zero MCC values by InterPro, due to the fact that those GO terms were not assigned to any *Drosophila* proteins or functional descriptions in the database. For predicting biological process terms, FFPred-fly+ obtains higher MCC values for 150 out of 196 terms (131 of them by more than 0.1) and 69 terms are not assigned to any *Drosophila* protein. For predicting molecular function terms, the InterPro-based method obtains higher MCC values for 50 out of 68 terms (44 by more than 0.1). For predicting cellular component terms, FFPred-fly+ obtains higher MCC values for 29 out of 37 terms (20 by more than 0.1) and InterPro has a zero MCC for 3 of them. The statistical significance test results further confirm that FFPred-fly+ performs better when predicting all 301 terms with p-value(All) = 4.4e-12, biological process terms with p-value(BP)<2.2e-16 and cellular component terms with p-value(CC) = 2.1e-05, while InterPro-based method performs better when predicting molecular function terms with p-value(MF) = 1.4e-06.

### Novel protein function prediction made by FFPred-fly+

To gauge the usefulness of the approach, we have looked at the novel GO term assignments made by FFPred-fly+ for proteins that currently have no meaningful biological process function annotations according to the latest UniProt-GOA database (version 10-04-2017). In detail, there are 4359 out of 19834 proteins (22%) that have only biological process root term annotations (i.e. GO:0008150), which convey no useful information, or don’t have any biological process term annotations at all. FFPred-fly+ assigned biological process annotations to 2964 (68%) of the unannotated proteins, with a high confidence (i.e. greater than 80% likelihood). For example, we report the prediction of GO:0030154 annotation for protein M9NEB4 with 90% confidence. All of our predictions are accessible via the following url: http://bioinfadmin.cs.ucl.ac.uk/ffpred/fly/.

## Discussion

Overall, as discussed in previous sections, the Random Forests (RF) method obtains the best performance when predicting function for the independent 30% protein set. The model learned by RF is also interpretable for mining meaningful patterns in the data itself. Therefore, in this section, we illustrate our model’s capacity to reveal links between certain protein functions and the developmental stages of *Drosophila* by analysing the relative importance of time-course expression-based features.

### Interpreting the importance of expression-based features for predicting biological process function

The importance of features indicates the power of the features for predicting target classes by the given classification algorithm. For Random Forests, the feature importance is evaluated by bootstrap analysis of the trained model. As shown in Eq 1, the feature importance FI_*f*_ is defined as the decrease on impurity (the mean decrease on Gini index between parent node to direct descendent nodes) among all contained trees (${\text{DecImp}}_{f}^{t}$) times the proportion of instances Prop(Inst_*f*_) used to construct the trees during the bootstrapping process, then normalised by the total number of trees (*No*. Tree) in the Random Forests.

$$\begin{array}{c} FI_{f}=\frac{\sum DecImp_{f}^{\tau }\times Prop\left(Inst_{f}\right)}{No.\;Tree} \end{array}$$ (1)

$$\begin{array}{c} RFI_{f}=\sqrt{\frac{FI_{f}}{FI_{Max\_global}}\times \frac{FI_{f}}{FI_{Max\_local}}} \end{array}$$ (2)

Note that, the best-performing types of features used for predicting BP, MF and CC terms are combinations of expression and sequence-based features. Therefore, we discuss the feature importance of expression-based features by considering two factors, i.e. the relative importance w.r.t. the feature having the global maximum importance among all expression-based and sequence-based features, and the relative importance w.r.t. the feature having the local maximum importance only within the expression-based features. As shown in Eq 2, we calculate the relative importance by obtaining the square root of the product of the proportion of individual feature importance against the global maximum feature importance and the local maximum feature importance respectively. The maximum value of RFI is 1.0, indicating that the feature has both globally and locally maximum importance value. In addition, recall that, the optimal types of feature combinations consist of three or two types of expression-based features (i.e. Seq+Num+Ave+Main for BP and MF terms, Seq+Ave+Main for CC terms). Therefore, for an individual feature (time-point), we only choose the feature having the maximum related importance. For example, in Seq+Num+Ave+Main types of feature, if the time-point 1 has 0.2 of importance in features Num, 0.5 of importance in features Ave, and 0.4 of importance in features Main, we select 0.5 as the importance value for time-point 1 feature of Seq+Num+Ave+Main. We then further group all 30 time-point features, by selecting the maximum feature importance among individual main developmental stages, i.e. time-points 0—12 for embryo, time-points 13—18 for larva, time-points 19—24 for pupa, and time-points 25—30 for adult.

**Fig 7:**
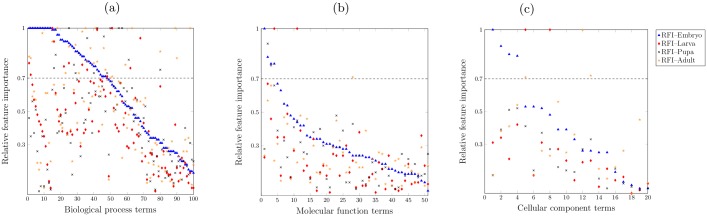
Distribution of related feature importance value for features denoting each of main developmental stages for *Drosophila*. (a) the distribution of RFI values for predicting biological process domain of GO terms; (b) the distribution of RFI values for predicting moleulcar function domain of GO terms; (c) the distribution of RFI values for predicting cellular component domain of GO terms.

We focus on the GO terms that obtain the highest accuracy by Random Forests when evaluating on the 30% test protein set (i.e. 100 BP terms, 51 MF terms, and 20 CC terms). The distribution of RFI values for all those GO terms is shown in Fig 7, where each colour of dots indicates the maximum relative feature importance value for each main developmental stage of *Drosophila melanogaster*.

Generally, the majority of expression-based features indicate high importance when predicting biological process functions. As shown in Fig 7.a, 61 out of 100 BP terms are with the RFI value being greater than the 0.7 of threshold at least one developmental stage. On the contrary, only a few expression-based features show high importance when predicting MF and CC terms. As shown in Fig 7.b and 7.c, almost all MF and CC terms have RFI<0.7 on all main developmental stages. These results further confirm the findings discussed in the previous sections, i.e. transcription expression profile-based features only show the relevance to predict the biological process function. Therefore, hereafter, we only further discuss the expression-based features’ relative importance on predicting biological process function.

### Identifying biological processes associated with certain *Drosophila melanogaster* developmental stages

**Fig 8:**
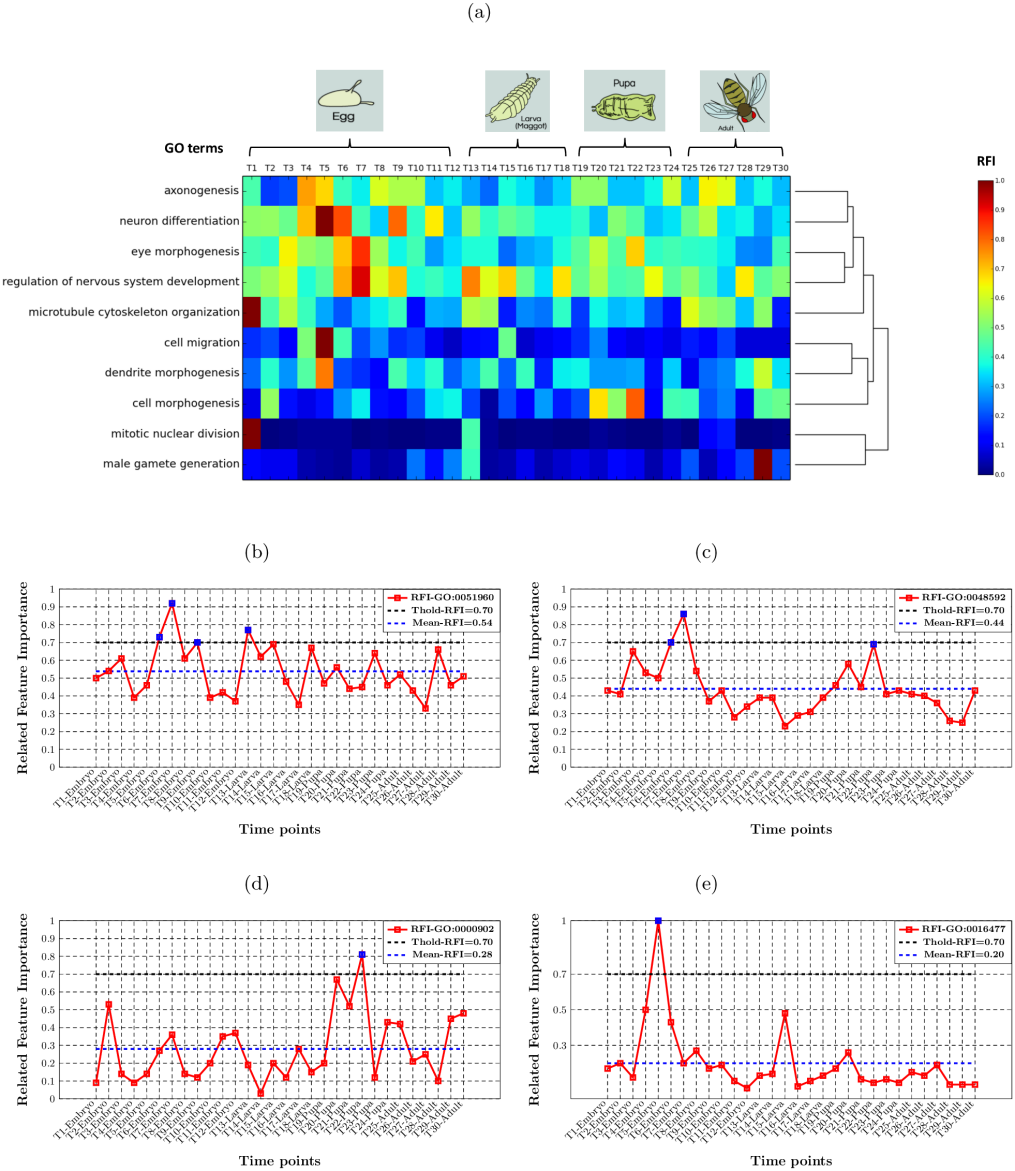
The distribution of RFI values for predicting 10 specific development-associated GO terms. (a) The heatmap of relative feature importance on predicting development-related BP terms by Random Forests classification algorithm; (b) regulation of nervous system development (GO:0051960); (c) eye morphogenesis (GO:0048592); (d) cell morphogenesis (GO:0000902); (e) cell migration (GO:0016477).

We display the relative feature importance values of 30 time-point features for predicting 10 types of specific development-associated biological process function in the heatmap shown in Fig 8.a. The reddest colour indicates the highest RFI value (i.e. 1.0), while the bluest colour denotes the lowest RFI value (i.e. 0.0). We also further conduct hierarchical clustering analysis on those 10 functions, according to their RFI values over 30 time-point features. Note that, we show the distribution of RFI values for all other 51 BP terms in the S1 Table. Those terms are either ancestor terms for those 10 specific terms or located in the Gene Ontology hierarchy with less than three ancestor layers (only denoting relative generic protein function).

In general, the analysis of RFI values of expression-based features successfully identifies the association between developmental processes and certain developmental stages. It is obvious that those clustered 4 functions in the top of the heatmap are all relevant to *Drosophila melanogaster*’s nervous system development. According to the heatmap, those functions indicate their high relevance with more than one developmental stage of *Drosophila melanogaster*.

Regulation of nervous system development (GO:0051960) shows an active role at the embryonic and larval stage of *Drosophila melanogaster* development. In Fig 8.b, the first peak of RFI value reaches 0.92 at T7-Embryo, along with the highest RFI value being obtained by T13-Larva. It is known that the formation of the central nervous system (CNS) starts at the early embryonic stage, in which the region of neuroectodermal cells are determined in order to form the neuroblasts with the delamination process from the epithelium at later embryonic stage [24–26]. At larval stage, the CNS vigorously develops as a process of neuronal morphogenesis and many adult-specific neurons are also produced with the neuroblasts persisting into larval life [27, 28]. The peripheral nervous system (PNS) is also active during embryonic and larval stages, in which the sensory neurons are stereotyped positioned, associating with many biological processes, such as asymmetric cell divisions and neighbouring neurons interactions [29].

Eye morphogenesis (GO:0048592) is another developmental process that is relevant to more than one main developmental stage. According to Fig 8.c, the RFI value reaches its peak value at T7-Embryo, and raises up again at T22-Pupa. It indicates the high relevance of eye morphogenesis on the embryonic and pupal stages. It has been known that the morphogenesis of *Drosophila* eyes commences with the development of eye anlage at the embryonic stage. The eye anlage is generated after the partition on dorsal head neuroectoderm [30], and then progresses to be the visual primordium that further derives to the eye-antennal imaginal disc [31]. The formation of adult eye is mainly progressed during the pupal stage, including the events related with the progression of morphogenetic furrow and pattern formation [32]. Note that, the pupal stage is also an especially important stage for the tissue morphogenesis of *Drosophila*, such as wing and leg morphogenesis [33, 34]. As shown in Fig 8.d, the peak of RFI value for predicting cell morphogenesis (GO:0000902) is obtained by the time-point T22-Pupa.

The distribution of RFI values for other functions listed at the bottom of the heatmap shows their relevance to unique developmental stages of *Drosophila*, such as cell migration, male gamete generation and so on. Cell migration (GO:0016477) is one function that is detected by our system as a typical biological process happening on the embryonic stage of *Drosophila*. As shown in Fig 8.e, it is clear that the RFI value reaches the peak of 1.0 at T5-Embryo, and it is obviously higher than the average RFI value of 0.20. Actually, cell migration is a well-studied area in biology, especially for the *Drosophila melanogaster* system, since it was found as a complex phenomenon during embryonic stage associates with the body plan of *Drosophila* [35–37]. This indicates significant cell movement activities, such as primordial germ cells (PGCs), phagocytic cells, cells of the tracheal system, etc. PGCs is a well-known group of cells that shape the gonads of *Drosophila*. During the germband extension process of the embryo, the PGCs are moved along the dorsal and then towards the center. After contacting with the somatic cells, the PGCs form the gonad on either side of embryo, during the retraction process of the germband.

In this study, we evaluate the predictive power of temporal transcription expression profiles for the task of novel *Drosophila melanogaster* protein function prediction. We conclude that the features generated based on expression profiles obtain better performance on predicting biological process function, whilst also indicating similar performance for predicting other domains of protein function, compared with conventional sequence-based features. Furthermore, by combining expression-based and sequence-based features, the performance for predicting all three domains of protein function can be further improved. Based on the optimal types of feature combinations, our newly-proposed protein function prediction approach also significantly outperforms FFPred-fly. With the help of our machine learning models, we further illustrate the capacity of our system to help highlight some of the links between protein function and developmental stages of *Drosophila melanogaster*.

## Materials and methods

### Protein sets generated for Gene Ontology term prediction

As our reference data set, we make use of all available *Drosophila melanogaster*-specific proteins and their corresponding Gene Ontology (GO) annotation data as described below. The Gene Ontology terms are categorised into the usual three domains of protein function, i.e. Biological Process (BP), Molecular Function (MF), and Cellular Component (CC). We firstly generate the protein sets for GO terms by adopting the consistent procedure described in [2]. In general, for each individual GO term (a.k.a. protein function), a set of proteins is grouped as one dataset, according to the annotation information. In that dataset, an instance (protein) is characterised by a set of features, and assigned to a class, i.e. either annotated with that GO term or non-annotated with that GO term. The total 258 features generated from the protein sequence information are the same as the ones used by [2], such as amino acid composition, transmembrane segments and so on (the full list of sequence-based features is included in Table E in S1 Text). Then the whole protein set is randomly divided into two subsets, with a proportion of 7:3. For cross-validation, 70% of proteins are used for classifier training, and the remaining 30% used for evaluating the performance of the trained classifier. The same 30% test set is also to compare the performance of our newly proposed *Drosophila melanogaster*-specific protein function prediction method with our previous sequence-based method [2] retrained on *Drosophila melanogaster*.

The annotations for *Drosophila melanogaster* proteins were retrieved from the Gene Ontology Annotation (GOA) for *Drosophila* [38] database (version 01-09-2014). The hierarchical dependency information between terms was retrieved from the Gene Ontology database [39] (version 12-09-2014). The amino acid sequences information was obtained from the UniProt Knowledgebase [40] (version 2014_08). In total, 10519 proteins are assigned to 301 GO terms, including 196 Biological Process terms, 68 Molecular Function terms, and 37 Cellular Component terms (see S2 Table).

### Evaluating the predictive power of time-course transcription expression profiles

We evaluate the predictive performance of transcription expression profile-based features in two ways. Firstly, we evaluate the predictive power of the newly-generated expression-based features by comparing with the predictive power of the conventional protein sequence-based features (i.e. Seq), due to its proven success on predicting different eukaryotic organisms’ protein-GO term annotations [2]. We further generate four more types of expression-based features, as the different combinations of those three generated types of expression-based features, i.e. Num+Ave, Num+Main, Ave+Main or Num+Ave+Main. All those 7 types of expression-based features plus Seq type of features are compared. The number of features in those 7 different types of features ranges from 30 to 258. For example, the number of features in Ave type of features is 30, denoting the average expression profile over 30 different time-points, and the total number of the conventional Seq type of features is 258.

Moreover, we further evaluate the predictive power of features that simultaneously consist of both expression-based and sequence-based features. We combine the Seq type of features with all other types of expression-based features (i.e. Seq+Num, Seq+Ave, Seq+Main, Seq+Num+Ave, Seq+Num+Main, Seq+Ave+Main, Seq+Num+Ave+Main), while still choosing the predictive performance of Seq features as the benchmark. The number of features in those types of features ranges from 258 to 348. For example, the Seq+Num+Ave+Main type of features includes 258 sequence-based features plus 3 types of expression-based features, while each type of those expression-based features consisting of 30 individual features.

### Evaluating the predictive performance of our newly proposed *Drosophila melanogaster*-specific protein function prediction method

We evaluate the predictive performance of this newly proposed *Drosophila melanogaster*-specific protein function prediction method by benchmarking with the standard FFPred 2.0 method [2]. In this work, we re-train FFPred 2.0 (hereafter denoting FFPred-fly) by training on the same *Drosophila melanogaster*-specific source data described in the previous section. In detail, we re-train the SVM classifiers for GO terms by adopting the 70%-split *Drosophila melanogaster* protein training set, then evaluate the performance on predicting the GO terms annotation for the remaining 30%. Note that, the grid-search hyper-parameter optimisation and the backwards feature group elimination processes are also included during the classifier training.

In this work, we choose the well-known Matthews Correlation Coefficient (MCC) (Eq 3) and the Area Under Receiver Operating Characteristics Curve (AUROC) as the metrics of predictive performance [2, 3]. The value of MCC ranges between -1 to 1, where value 0 indicates that the predictive performance is not better than random prediction. The value of AURCO ranges between 0.5 to 1.0, where 0.5 denotes the random predictive performance and 1.0 denotes the perfect predictive performance.

$$\begin{array}{c} \text{MCC}=\frac{TP\times TN-FP\times FN}{\sqrt{\left(TP+FP)(TP+FN)(TN+FP)(TN+FN\right)}} \end{array}$$ (3)

## Supporting information

S1 Text Supporting text. (PDF)

S1 Table Relative feature importance (RFI) values for 61 biological process terms that are with at least one time-point’s RFI value being greater than 0.7 of threshold. (XLS)

S2 Table List of Gene Ontology terms studied in this work. (XLS)
