# Supplementary material for: Analysis of temporal transcription expression profiles reveal links between protein function and developmental stages of Drosophila melanogaster
Source: PLoS Comput Biol. 2017 Oct 18;13(10):e1005791. doi: 10.1371/journal.pcbi.1005791 (PMC5662236; doi:10.1371/journal.pcbi.1005791)
Supplement: S1 Text — (PDF) [file pcbi.1005791.s001.pdf]

Supporting Text

Analysis of temporal transcription expression profiles reveal links between protein function and developmental stages of *Drosophila melanogaster*

Cen Wan [1,3], Jonathan G. Lees [2], Federico Minneci [1], Christine A. Orengo [2] and David T. Jones [1,2,3]

[1]Department of Computer Science, University College London, London, United Kingdom, [2]Institute of Structural and Molecular Biology, University College London, London, United Kingdom and [3]The Francis Crick Institute, London, United Kingdom

The Supporting Information includes

Description of classification algorithms adopted in this work

Significance test results (Tables A and B)

The optimal types of features for predicting three domains of GO terms (Figures A and B, Tables C and D)

The best performing classification algorithm for predicting GO terms (Figures C and D)

List of sequence-based features (Table E)

Description of Classification algorithms adopted in this work

We use four types of classification algorithms, i.e. Random Forests (RF), Adaptive-Boosting (AdaBoost), k-Nearest Neighbours (KNN) and Linear Discriminant Analysis (LDA), to evaluate the predictive performance of different types of features for predicting proteins' GO terms annotation. All those classification algorithms were implemented by the Scikit-learn machine learn library.

The first classification algorithm, i.e. Random Forests, is a well-known ensemble learning-based classification algorithm. It infers the classification according to the averaged classification decision made by a set of decision tree classifiers. Those different decision tree classifiers are learnt from different training datasets sampled by bootstrapping (with replacement) strategy. In this work, we tune four parameters of Random Forests classification algorithm, i.e. the number of trees included in the forest, the maximum of depth on the individual trees, the maximum number of features being considered as the split point on the trees, and the metrics of measuring the features' predictive power.

Adaptive-Boosting is a type of meta-learning classification algorithm, which sequentially adds more weak classifiers into the decision making process, by simultaneously adjusting the weights of training instances. The weights of instances that are increased denotes those instances are incorrectly classified on previous iteration. Therefore, after several times of iteration, the classifiers will more focus on those incorrectly classified instances. In this work, we choose decision stump as the base classifier and tuned two parameters of Adaptive-Boosting classification algorithm, i.e. the number of base classifiers, and the learning rate.

K-nearest neighbours method is a type of instance-based classification algorithm. Instead of building a general model for the whole training dataset, KNN predicts the class label of individual testing instance by considering its corresponding k closest neighbours' class labels. The decision of classification is made by voting mechanism, which means the class label belongs to the majority of neighbouring instances is assigned as the class label of that testing instance. In this work, we use two different strategies to consider the neighbouring instances for individual testing instance. The first metric is that all neighbouring instances are assigned as equal weight, without considering the actual distance to the testing instance. The second metric is that all neighbouring instances are assigned with a weight which is the inverse of the distance to the testing instance. It means that the class label of closer neighbouring instance has higher probability to be assigned as the class label for the testing instance.

Linear discriminant analysis is a type of supervised learning-based dimensionality reduction method. On the training stage, it searches for the linear boundary in the space with low dimensionality, while the projection of instance on that boundary having the maximum variance between classes and minimum scatter degree within individual classes. LDA method has the advantages on low computational cost and parameter-free.

Significance test results

Table A. Wilcoxon signed-rank test results on predictive performance (based on MCC value) of different types of expression-based features comparing with sequence-based features

Domain	Benchmark	Num	Ave	Main	Num+Ave	Num+Main	Ave+Main	Num+Ave+Main
BP	Seq	**-**	**+**	**+**	**+**	**null**	**+**	**+**
MF		**-**	**-**	**-**	**-**	**-**	**-**	**-**
CC		**-**	**null**	**null**	**null**	**-**	**null**	**null**

+: that type of expression-based features significantly outperforms sequence-based features;
-: the sequence-based features significantly outperform that type of expression-based features;
null: there is no significant difference between two types of features' performance

Table B. Wilcoxon signed-rank test results on predictive performance (based on AUROC value) of different types of expression-based features comparing with sequence-based features

Domain	Benchmark	Num	Ave	Main	Num+Ave	Num+Main	Ave+Main	Num+Ave+Main
BP	Seq	**-**	**+**	**+**	**+**	**+**	**+**	**+**
MF		**-**	**-**	**-**	**-**	**-**	**-**	**-**
CC		**-**	**null**	**-**	**null**	**-**	**null**	**null**

+: that type of expression-based features significantly outperforms sequence-based features;
-: the sequence-based features significantly outperform that type of expression-based features;
null: there is no significant difference between two types of features' performance

The optimal types of features for predicting three domains of GO terms

We compare the predictive performance of all 15 different feature types over the cross validation process and held-out set evaluation. The distribution of MCC and AUROC values for different types of features obtained by Opt-Classifier when predicting three domains of GO terms are shown in the boxplots in Figures S1 and S2. We also report the ranking of predictive performance for different types of features for predicting three domains of GO terms in Tables S3 and S4.

Figures S1(a,c,e) and Figures S2(a,c,e) show the MCC and AUROC values obtained during cross validation over 70% of protein set, while Figures S1(b,d,f) and Figures S2(b,d,f) show the MCC and AUROC values obtained by adopting 70% of training protein set and evaluating on 30% of independent protein set.

In each boxplot, the left and right boundaries respectively denote the lower and upper quartile of a distribution of MCC or AUROC values. The vertical line indicates the medium MCC or AUROC value, while the red diamond denotes the mean MCC or AUROC value. The horizontal lines extend from the left and right boundaries of a box denote the lowest and highest non-outlier MCC or AUROC values. The out-outlier MCC or AUROC values

are shown as the circle points. Note that, in each boxplot, the boxes are sorted according to their mean MCC or AUROC values, i.e. the box with the lowest mean MCC or AUROC value is at the bottom of figure.

For predicting biological process domain of GO terms, as shown in Figures S1(a,b) and S2(a,b), almost all combinations of expression-based and sequence-based features (except *Seq+Num*) obtain higher mean and third quartile MCC and AUROC values than the ones obtained by different types of expression-based only features both on cross validation and evaluation on 30% of protein set. Among all different types of features, *Seq+Num+Ave+Main* obtains the best ranking over cross validation (i.e. ranking of 2) and held-out set evaluation (i.e. ranking of 2).

For predicting molecular function domain of GO terms, as shown in Figures S1(c,d) and S2(c,d), all types of feature combinations obtain the higher mean and third quartile MCC and AUROC values than the ones obtained by expression-based type of features. Feature combination of *Seq+Num+Ave* and *Seq+Ave+Main* obtains the best ranking over cross validation (i.e. ranking of 3), while the feature combination of *Seq+Num+Ave+Main* obtains the best ranking over held-out set evaluation (i.e. ranking of 3).

Analogously, when predicting cellular component domain of GO terms, as shown in Figures S1(e,f) and S2(e,f), all feature combinations obtain higher medium, mean and third quartile MCC values than the ones obtained by all types of expression-based features. Feature combination *Seq+Ave+Main* obtains the best best ranking over cross validation (i.e. ranking of 2) and held-out set evaluation (i.e. ranking of 5).

Therefore, it can be concluded that feature combination *Seq+Num+Ave+Main* performs best when predicting biological process domain of GO terms both on cross validation and the evaluation on 30% of protein set. It also performs best on predicting molecular function domain of GO terms when evaluation on 30% of protein set. Feature combination *Seq+Ave+Main* performs best on predicting cellular component domain of GO terms, and on predicting molecular function domain of GO terms over cross validation.

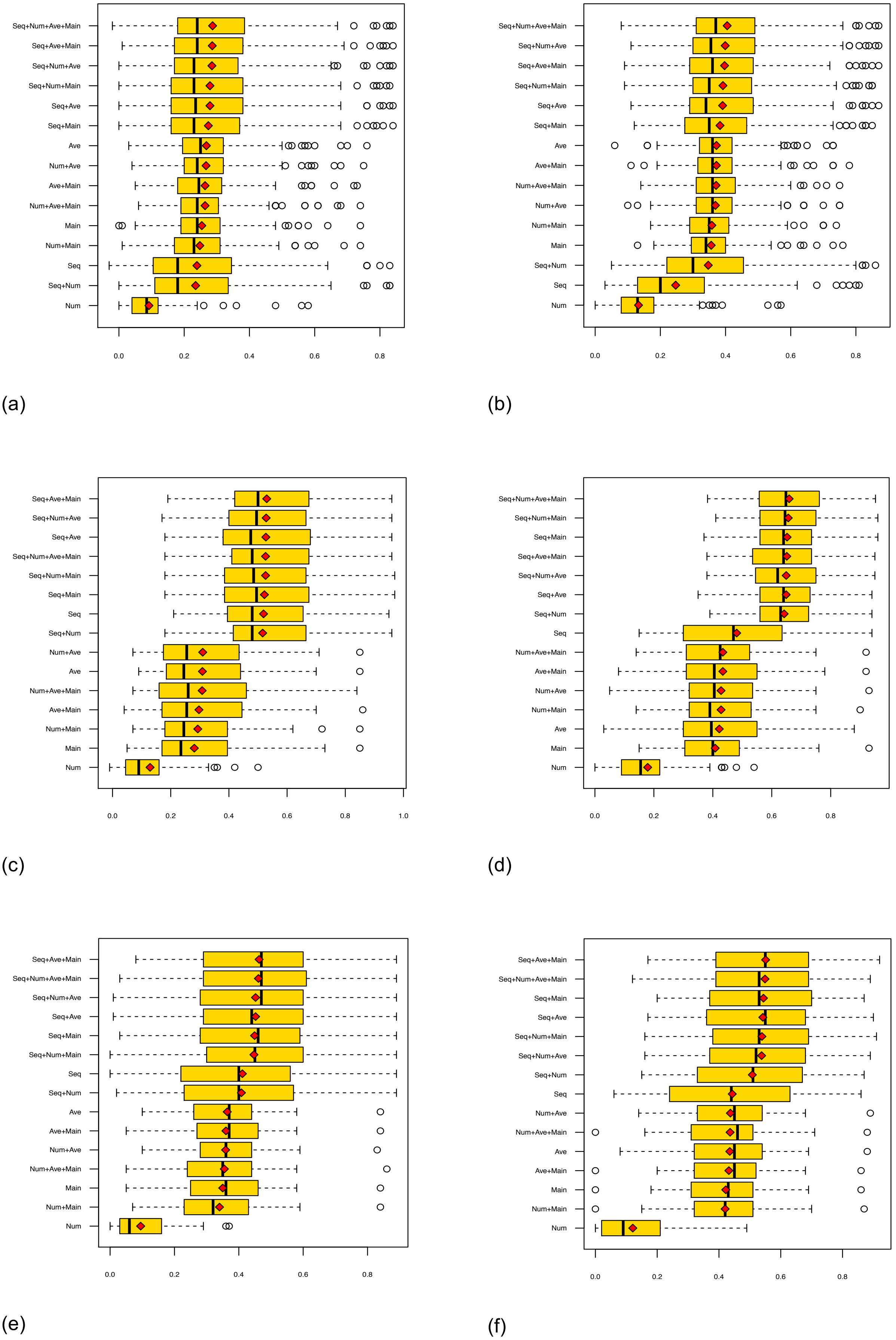

Figure A. Boxplots about distribution of MCC values obtained by all 15 different types of features during cross validation and the evaluation on 30% of protein set, for predicting BP (a,b), MF (c.d) and CC (e.f) terms.

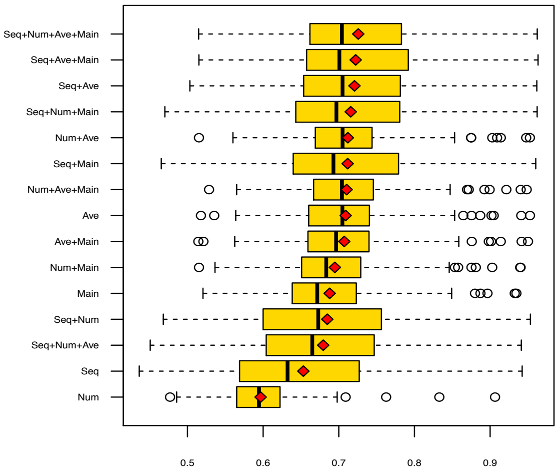

(a)

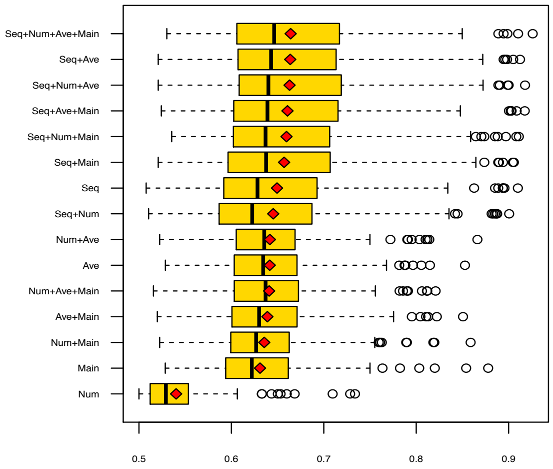

(b)

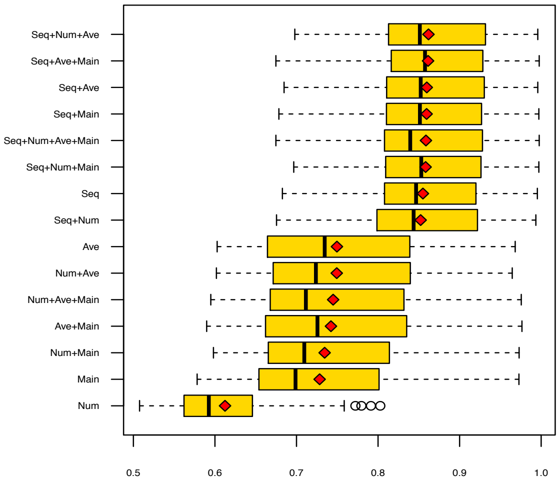

(c)

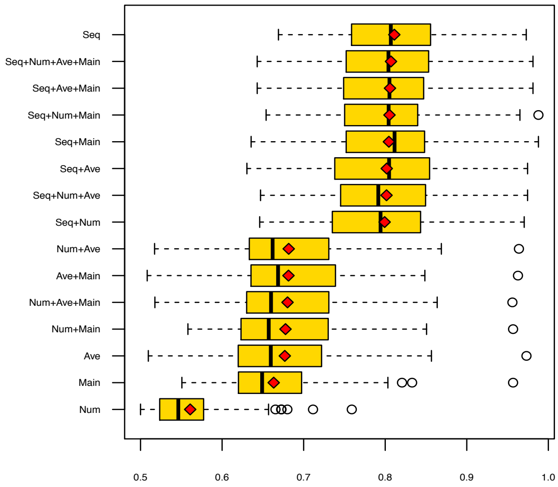

(d)

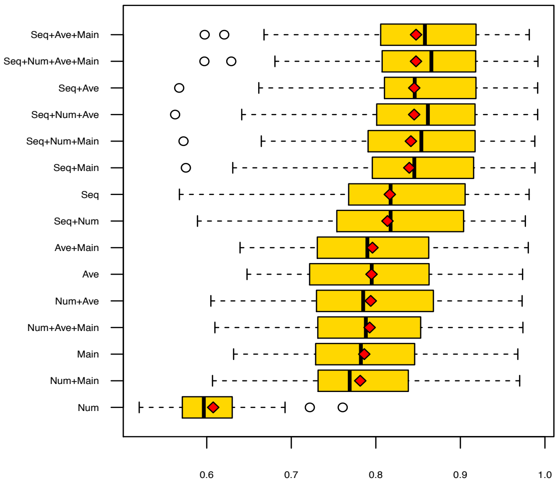

(e)

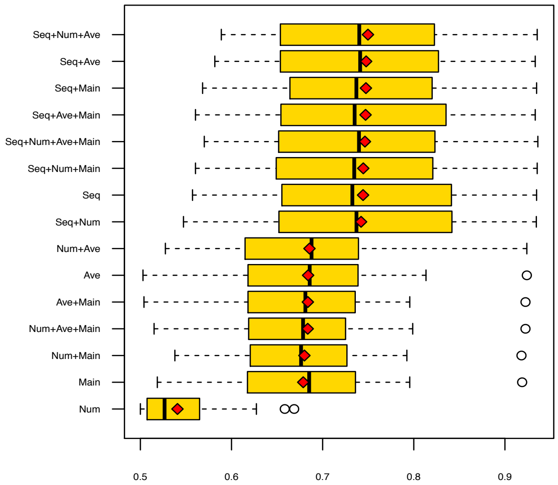

(f)

Figure B. Boxplots about distribution of AUROC values obtained by all 15 different types of features during cross validation and the evaluation on 30% of protein set, for predicting BP (a,b), MF (c,d) and CC (e,f) terms.

Table C. Ranking of predictive performance obtained by different feature groups on predicting three domains of protein function over the cross validation on 70% of protein set

70% Cross Validation											
	BP				MF				CC		
**Ranking**	**MCC**	**AUROC**	**Sum**		**MCC**	**AUROC**	**Sum**		**MCC**	**AUROC**	**Sum**
Num	15	15	30		15	15	30		15	15	30
Ave	7	8	15		10	9	19		9	10	19
Main	11	11	22		14	14	28		13	13	26
Num+Ave	8	5	13		9	10	19		11	11	22
Num+Main	12	10	22		13	13	26		14	14	28
Ave+Main	9	9	18		12	12	24		10	9	19
Num+Ave+Main	10	7	17		11	11	22		12	12	24
Seq	13	14	27		7	7	14		7	7	14
Seq+Num	14	12	26		8	8	16		8	8	16
Seq+Ave	5	3	8		3	3	6		4	3	7
Seq+Main	6	6	12		6	4	10		5	6	11
Seq+Num+Ave	3	13	16		2	1	**3**		3	4	7
Seq+Num+Main	4	4	8		5	6	11		6	5	11
Seq+Ave+Main	2	2	4		1	2	**3**		1	1	**2**
Seq+Num+Ave+Main	1	1	**2**		4	5	9		2	2	4

Table D. Ranking of predictive performance obtained by different feature groups on predicting three domains of protein function over the 30% of held-out protein set evaluation

30% Held-out set Evaluation											
	BP				MF				CC		
**Ranking**	**MCC**	**AUROC**	**Sum**		**MCC**	**AUROC**	**Sum**		**MCC**	**AUROC**	**Sum**
Num	15	15	30		15	15	30		15	15	30
Ave	7	10	17		13	13	26		11	10	21
Main	12	14	26		14	14	28		13	14	27
Num+Ave	10	9	19		11	9	20		9	9	18
Num+Main	11	13	24		12	12	24		14	13	27
Ave+Main	8	12	20		10	10	20		12	11	23
Num+Ave+Main	9	11	20		9	11	20		10	12	22
Seq	14	7	21		8	1	9		8	7	15
Seq+Num	13	8	21		7	8	15		7	8	15
Seq+Ave	5	2	7		6	6	12		4	2	6
Seq+Main	6	6	12		3	5	8		3	3	6
Seq+Num+Ave	2	3	5		5	7	12		6	1	7
Seq+Num+Main	4	5	9		2	4	6		5	6	11
Seq+Ave+Main	3	4	7		4	3	7		1	4	**5**
Seq+Num+Ave+Main	1	1	**2**		1	2	**3**		2	5	7

The best performing classification algorithm for predicting GO terms

In terms of the comparison on predictive performance obtained by different classification algorithms, Figures S3 and S4 display the proportion of GO terms selecting different single classification algorithms as the Opt-Classifier working with the corresponding optimal types of feature groups, respectively on cross validation and the evaluation on 30% of independent protein set, based on the metrics of MCC and AUROC. Obviously, for predicting the biological process domain of GO terms, K-Nearest Neighbours and Random Forests classification algorithms perform best. KNN obtain the highest MCC and AUROC values on 40.8% and 40.3% of GO terms over cross validation, while 31.6% and 38.8% of GO terms over the held-out set evaluation. RF obtains the highest MCC and AUROC values on 38.3% and 51.0% of GO terms over cross validation, and 51.0% and 23.5% of GO terms over the held-out set evaluation.

For predicting the molecular function domain of GO terms, RF obtains the best MCC values on 45.6% and 75.0% of GO terms, while also obtains the best AUROC values on 79.4% and 47.1% of GO terms, over cross validation and the evaluation on 30% of independent protein set respectively.

Analogously, for predicting the cellular component domain of GO terms, RF obtains the highest MCC values on 40.5% and 54.1% of GO terms, over cross validation and the evaluation on 30% of independent protein set, respectively. It also obtains the highest AUROC values on 81.1% of GO terms over cross validation and the second highest AUROC on 24.3% of GO terms.

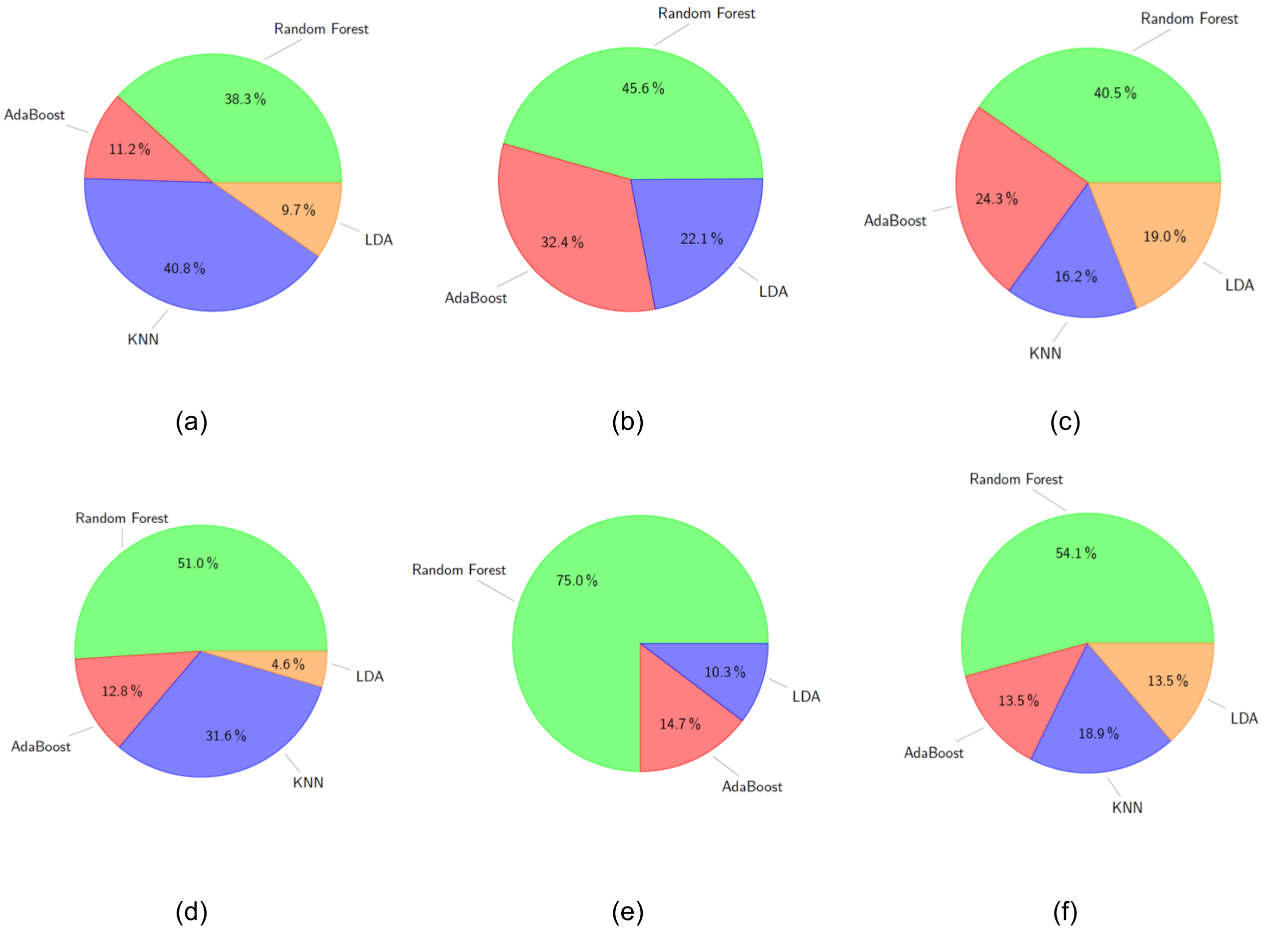

Figure C. The proportion of GO terms for which different classification algorithms being selected as the Opt-Classifier during the cross validation and the evaluation on 30% of protein set, based on metric of MCC value, for predicting BP (a,d), MF (b,e) and CC (c,f) terms.

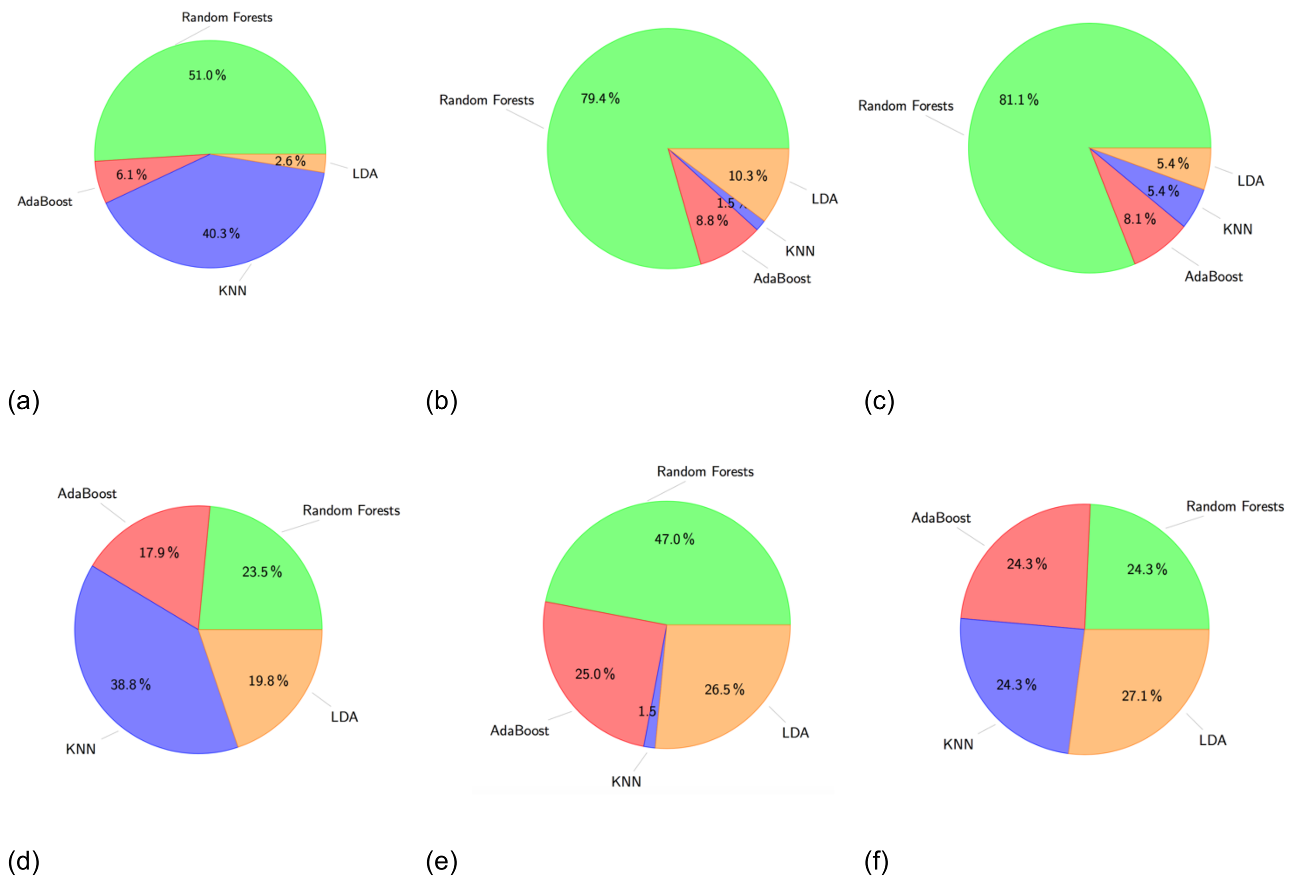

Figure D. The proportion of GO terms for which different classification algorithms being selected as the Opt-Classifier during the cross validation and the evaluation on 30% of protein set, based on metric of AUROC value, for predicting BP (a,d), MF (b,e) and CC (c,f) terms.

Table E. List of sequence-based features

**Feature name**
Secondary structure
Transmembrane segments
Amino acid composition
Intrinsically disordered regions
Signal peptides
Subcellular localization
Sequence features
PEST regions
Low complexity regions

Coiled coils
N-linked glycosylation sites
O-GalNAc-glycosylation sites
Phosphorylation sites
